# Effect of pachymaran on oxidative stress and DNA damage induced by formaldehyde

**DOI:** 10.1038/s41598-023-44788-y

**Published:** 2023-10-14

**Authors:** Zhijun Zhang, Yuan Yang, Changjun Hu, Zaiqi Zhang

**Affiliations:** 1 College of Public Health and Laboratory Medicine, Hunan University of Medicine, 492 Jinxi South Road, Huaihua, Hunan 418000 People’s Republic of China; 2 Hunan Provincial Key Laboratory of Dong Medicine, Hunan University of Medicine, Huaihua, Hunan 418000 People’s Republic of China

**Keywords:** Biomarkers, Medical research, Molecular medicine

## Abstract

To further explore the pharmacological effect of pachymaran, this article studied the inhibition of pachymaran on oxidative stress and genetic damage induced by formaldehyde. 40 adult Kunming male mice were randomly divided into four groups with different interventions. One week later, the contents of serum SOD, GR, MDA, DNA–protein crosslink (DPC), 8-hydroxydeoxyguanosine (8-OHDG) and DNA adduct were determined by ELISA. The results showed that there were statistically significant differences in the contents of SOD, GR and MDA among the four groups (*P* < 0.01). The activity of SOD and GR increased along with the increase of pachymaran dosage (SOD: r_s_ = 0.912, *P* < 0.01; GR: r_s_ = 0.857, *P* < 0.01), while the content of MDA showing a significant negative correlation (r_s_ = − 0.893, *P* < 0.01). There were statistically significant differences in the levels of DPC, 8-OHDG and DNA adduct among the four groups (DPC and DNA adduct: *P* < 0.01, 8-OHDG: *P* < 0.05), the concentration decreased along with the increase of pachymaran dosage (DPC: r_s_ = − 0.855, *P* < 0.01; 8-OHDG:r_s_ = − 0.412, *P* < 0.05, DNA adduct: γ_s_ = − 0.869, *P* < 0.01). It can be inferred that pachymaran can inhibit oxidative stress and DNA damage induced by formaldehyde with the dose–effect relationship.

## Introduction

Poria cocos is a dry sclerotium of the genus lycoris of porous fungus family. It tastes sweet and mild, and has the function of invigorating spleen, nourishing heart, tranquilization, and relieving diuresis and moisture penetration. In Asian countries such as China, South Korea, Japan and Thailand, it have a long history of medicinal poria cocos. As a dual-use fungus, its chemical components mainly include triterpenoids, polysaccharides, steroids, amino acids, choline, etc. Poria cocos is an important traditional Chinese medicine, which can play its role in combination with many medicinal herbs. Pachymaran is a bioactive substance extracted from poria cocos, which is composed of glucose, galactose, xylose, mannose and other components, and has pharmacological effects of anti-tumor, anti-aging, lowering blood lipids, anti-virus, anti-inflammatory and immune regulation^1–3^. Some experiments have proved that pachymaran can significantly increase the thymus index of immunosuppressed mice induced by cyclophosphamide, reduce the level of interleukin-10 (IL-10) in mice, enhance the phagocytosis of peritoneal macrophages in mice, increase the level of immunoglobulin G (IgG) and immunoglobulin M (IgM) antibodies in mice, and alleviate the inhibition of cyclophosphamide on the humoral immune function of mice^4^. The polysaccharide formula containing pachymaran can improve the immune activity by restoring the expression of T cell immune regulatory genes cell adhesion molecule1 (CADM1), chemokine C-C-motif receptor 2 (CCR2), immunoglobulin lambda like polypeptide1 (IGLL1), interferon inducible GTPase 1 (LIGP1), Fc-gamma RIII (FCGR3) and Fc-gamma RII (FCGR2) and B cell immune regulatory genes S100 calcium binding protein A8 (S100A8), S100 calcium binding protein A9 (S100A9), chitinase-like 3 (ChIL3), myeloid-related protein-8 (MMP8) and interferon-induced transmembrane protein3 (IFITM3) in immunosuppressed mice^5^. Pachymaran plays also a regulatory role against macrophages by toll-like receptor 4 (TLR4)/tumor necrosis factor receptor-associated factor 6 (TRAF6)/nuclear factor kappa-B (NF-κB) and protein kinase C (Ca^2+^/PKC)/nuclear factor-kappa B (p38/NF-κB) signal pathway^6,7^. Carboxymethylated pachymaran (CMP) can significantly reduce the content of MDA in male BALB/c mice, and significantly increases the levels of serum hemolysin antibody titer and the spleen antibody^8^.

Research has found that CMP slows down the decreased transmembrane resistance and the increased phenol red flux induced by tumor necrosis factor (TNF-α), and inhibits TNF-α induced expression of myosin light chain kinase, and inhibits TNF-α induced phosphorylation levels of myosin light chain, transcription factor protein (NF-κB) P65 and nuclear factor κB inhibitor protein inhibitor of NF-κB-α (IκBα), consequently leading to the reduced risk of inflammation^9^. CMP can regulate the ecological balance of intestinal flora in CT26 tumor-bearing mice induced by 5-fluorouracil, thus reduces intestinal injury^10^, and significantly improve the colitis of trinitrobenzene sulfonic acid (TNBS)-induced model mice, characterized as reducing mortality and down-regulating the level of pro-inflammatory cytokines in colon tissue and serum^11^. Pachymaran can reduce the activation of mast cells (MC) and inhibits the expression of inflammatory factors by the inhibition of IL-33(interleukin 33)/ST2(growth stimulating expression gene 2) signal pathway^12^.

Hou et al. found that pachymaran compound oral liquid can inhibit the growth of sarcoma 180 (S180) and hepatoma 22 (H22) tumors in mice, significantly enhances macrophage phagocytosis, promotes lymphocyte proliferation and NK cell activity in mice, and regulates the immune function of tumor-bearing mice^13^. Pachymaran can lead to the imbalance of (B-cell lymphoma-2 (Bcl-2)/Bcl2-associated X (Bax) by regulating the ratio of Bcl-2/Bax protein, inhibits the growth of tumor cells and induces apoptosis of human breast cancer cells by activating apoptosis signaling pathway^14,15^. Both CMP and pachymaran have certain inhibitory effects on the proliferation of HepG-2 cells^16^. Also, pachymaran inhibits significantly the proliferation and induces the apoptosis in HeLa cells^17^.

Whether the experiments in vivo or in vitro, pachymaran shows a good protective effect on acetaminophen (APAP)—induced hepatocytes injury, and the potential mechanism may be interpreted by inhibiting cell death, reducing the inflammatory stress of hepatocytes and inhibiting the biological activity of Hsp90(heat shock protein90)^18,19^. Some studies have found that CMP can increase significantly the index of thymus and spleen, enhance the activity of lysozyme and catalase, and inhibit the activity of xanthine oxidase^20^. In addition, pachymaran has a good anti-fatigue effect^21^.

DNA–protein crosslinks (DPC) is one of the important markers of genetic damage of biological macromolecules. Its formation can affect gene expression, break the normal structure of chromosome, and lead to the change and loss of some important genetic information in the process of DNA replication, thus inducing mutation and tumor. DNA adduct is one of covalent binding products formed by electrophilic compounds or their metabolites and DNA in body, and are the most important and common form of chemical DNA damage. At present, it is believed that the covalent combination of exogenous compounds and DNA may lead to gene mutations at some specific sites when the formed conjugates escape from DNA repair mechanism in body. Therefore, the formation of DNA adduct is considered to be an important stage in the tumorigenic process. DPC and DNA adduct can be used as molecular level exposure biomarkers to reflect the exposure dose of the poison to the target site, and as an effect marker to reflect the degree of DNA damage by toxic chemicals. Therefore, inhibiting the formation of DPC and DNA adduct shows a positive effect on preventing the occurrence of tumors and protecting the health of body.

The occurrence of aging, tumor, diabetes and many neurodegenerative diseases is related to oxidative damage. 8-hydroxy-2 deoxyguanosine (8-OHDG) is an oxidative adduct produced by reactive oxygen free radicals such as hydroxyl free radicals, singlet oxygen, which can attack the 8th carbon atom of guanine base in DNA molecules. Usually, it is used to assess the degree of oxidative damage and repair in body; Superoxide dismutase (SOD) is an important antioxidant enzyme that is widely distributed in various organisms, such as animals, plants, microorganisms, etc., and it is the primary substance for scavenging free radicals in organisms; Glutathione reductase (GR) is the main flavin enzyme that maintains the content of reduced glutathione (GSH) in cells. It plays an important role in preventing from the oxidative decomposition of hemoglobin, ensuring the reduction of sulfhydryl protein and retaining cell integrity; Malondildehyde (MDA) is the end product of lipid peroxidation reaction caused by free radicals, which can cause cross-linking polymerization of proteins, nucleic acids and other living macromolecules, and can lead to cellular toxicity.

## Materials and methods

### Main reagents and instruments

Pachymaran extract (Xi'an Kaikai Bioengineering Co., Ltd., containing pachymaran 63.5%, which is dissolved and diluted by physiological saline before use); Formaldehyde (analytically pure, prepared with physiological saline before use); ELISA kit for detection of SOD, GR and MDA (Jiangsu Kaiji Biotechnology Co., Ltd.); DPC, DNA adduct and 8-OHDG detection ELISA kit (Shanghai Yili Biotechnology Co., Ltd.); RT-6000 enzyme label analyzer (Shenzhen Redu Life Sciences Co., Ltd.); TD24-WS low-speed automatic balance centrifuge (Changsha Xiangyi Centrifuge Instrument Co., Ltd.).

### Experimental animals

This study was conducted in close conformity with the regulations of the Institutional Animal Care and Use Committee of Hunan University of Medicine (HNMU-IACUC), approval number(No.2022A0316). All methods in this study were carried out in accordance with ARRIVE guidelines.

40 adult healthy Kunming male mice were provided by the Experimental Animal Center of Xiangya Medical College, Central South University, with the license number of SCXK (Xiang) 2009-0002, the qualification number is 20-011, and the weight range is 20 ± 1.03 g. Before starting the experiment, the mice were first fed in a well-ventilated and well-lit laboratory with normal feed, free diet and water intake. The laboratory temperature was 18–24 °C, and the humidity was 30–40%. The mice were fed at 7 o'clock every night, and the water and bedding were changed every two days. The mice were used for the experiment after 4 days of feeding. The method and dose of formaldehyde exposure refer to the study of Ge et al.^22^, and the dose of pachymaran refer to the study of Cheng et al^23,24^.

### Experimental protocol

These mice were randomly divided into four groups: (1) formaldehyde exposure group (mice were given 0.4 mL of formaldehyde solution with a concentration of 0.5 g/L by oral administration); (2) low dose of pachymaran group (formaldehyde + 2 g/L pachymaran); (3) medium dose of pachymaran group (formaldehyde + 4 g/L pachymaran) and (4) high dose of pachymaran group (formaldehyde + 8 g/L pachymaran). Detailedly, In order to eliminate the impact of different gavage amounts on the results, we used the same amount of gavage in each group, so the concentration of formaldehyde solution used in the positive group was 0.5 g/L with a gavage volume of 0.4 mL, and the concentration of formaldehyde solution in pachymaran treatment group was 1.0 g/L with a gavage volume of 0.2 mL. In the low or medium or high dose of pachymaran group, mice were given 0.2 mL of formaldehyde solution with a concentration of 1.0 g/L by gavage and 0.2 mL of pachymaran solution with the different concentrations by gavage (2.0 g/L, low dose of pachymaran group; 4.0 g/L, medium dose of pachymaran group; 8.0 g/L, high dose of pachymaran group). The sample size was 10 in each group.

After 7 days of continuous gavage (one died in the middle and low dose groups of pachymaran), mice were anesthetized using pentobarbital 50 mg/kg via intraperitoneal injection. After that, eyeballs were taken to take blood, and the serum was centrifuged for 3 min at 1500 r/min, The activity of SOD and GR, the content of MDA,DNA adduct,DPC and 8-OHDG in serum of mice in each group were determined by ELISA. The activity of SOD and GR was expressed in U/mL, the content of MDA was expressed in nmol/mL, and the content of DNA adduct, DPC and 8-OHDG was expressed in ng/L. The specific operation was carried out according to the requirements of the kit operating instructions.

### Statistical treatment

The data were analyzed with ANOVA analysis, Spearman grade correlation, Kruskal Wallis Test of independent samples by *SPSS*21.0 statistical software, and *P* < 0.05 was considered as a significant difference.

### Ethical approval

The study was approved by the Institutional Animal Care and Use Committee, Hunan University of Medicine (No.2022A0316).

## Results

### Effect of pachymaran on SOD, GR and MDA

There were the significant differences in the contents of SOD, GR and MDA among the four groups (SOD: F = 45.821, *P* < 0.01; GR: F = 42.230, *P* < 0.01; MDA: F = 36.855, *P* < 0.01). when the dose of pachymaran was higher, the activity of SOD or GR was higher, showing a significant dose–effect relationship (SOD: Spearman grade correlation coefficient r_s_ = 0.912, *P* < 0.01; GR: Spearman grade correlation coefficient r_s_ = 0.857, *P* < 0.01). However, the level of MDA was negatively correlated with the dosage of pachymaran (Spearman grade correlation coefficient r_s_ = − 0.893, *P* < 0.01). This results suggested that pachymaran can increase the active levels of SOD and GR in formaldehyde exposure mice, and reduce the formation of MDA, more details were shown in Table 1.

**Table 1:** Inhibitory effect of pachymaran on formaldehyde-induced oxidative stress in mice(Mean ± SD $\overline{x} \pm s$).

Group	n	SOD(U/mL)	GR(U/L)	MDA(nmol/mL)
Positive control group	8	126.39 ± 14.61	18.38 ± 0.62	13.18 ± 0.67
Low-dose pachymaran + formaldehyde group	9	129.19 ± 11.99	19.92 ± 1.85^※^	12.70 ± 0.67
Medium-dose pachymaran + formaldehyde group	10	158.91 ± 9.81^※※^	21.96 ± 0.98^※※^	11.55 ± 0.49^※※^
High-dose pachymaran + formaldehyde group	10	180.78 ± 10.32^※※^	25.32 ± 1.70^※※^	9.87 ± 1.01^※※^

Compared with the positive control group,^※^*P* < 0.05, ^※※^*P* < 0.01.

There is a positive correlation between SOD and GR, both SOS and GR were negatively correlated with MDA, the details were shown in Table 2.

**Table 2:** Correlation among MDA and SOD and GR.

Correlation variable	*n*	coefficient	*P*
MDA/SOD	37	− 0.959	0.000
SOD/GR	37	0.850	0.000
GR/MDA	37	− 0.900	0.000

The results suggested that pachymaran increased the level of SOD and GR, reduced the formation of MDA, and showed its ability of resisting oxidative damage along with the increase of pachymaran dose.

### Effect of pachymaran on DPC, 8-OHDG and DNA adduct

There were significant differences in DPC and DNA adduct concentrations among the four groups (*Kruskal Wallis Test*, DPC: H = 27.551, *P* < 0.01; DNA adduct: H = 28.354, *P* < 0.01); the difference of 8-OHDG concentration among the four groups was also statistically significant (F = 4.59, *P* < 0.01). The concentration of DPC, 8-OHDG and DNA adduct was negatively correlated with the dose of pachymaran (DPC: *Spearman correlation coefficient* r_s_ = − 0.855, *P* < 0.01; 8-OHDG: rs = − 0.412, *P* < 0.05;DNA adduct: r_s_ = − 0.869, *P* < 0.01). more details were shown in Table 3.

**Table 3:** Inhibition effect of pachymaran on DPC, 8-OHDG and DNA adduct induced byformaldehyde ($\overline{x} \pm s$ Mean ± SD, ng/L).

Group	n	DPC	8-OHDG	DNA adduct
Positive control group	8	108.86 ± 14.01	59.46 ± 9.27	155.26 ± 33.98
Low-dose pachymaran + formaldehyde group	9	90.16 ± 4.41^※※^	53.29 ± 6.08^※^	134.93 ± 21.77
Medium-dose pachymaran + formaldehyde group	10	77.71 ± 4.77^※※^	50.24 ± 4.13^※※^	79.53 ± 11.05^※※^
High-dose pachymaran + formaldehyde group	10	72.80 ± 7.93^※※^	49.96 ± 4.67^※※^	63.75 ± 14.67^※※^

Compared with the positive control group, ^※^*P* < 0.01, ^※※^*P* < 0.01.

The positive correlations were observed among DPC, DNA adduct and 8-OHDG, the details were shown in Table 4.

**Table 4:** Correlation among DPC and 8-OHDG and DNA adduct.

Correlation variable	*n*	coefficient	*P*
DPC/8-OHDG	37	0.361	0.028
8-OHDG/DNA adduct	37	0.386	0.018
DNA adduct/DPC	37	0.634	0.000

## Discussion

Formaldehyde is a confirmed human carcinogen with genetic toxicity and widely existing in life and work environment. For example, artificial panels (large core panels, composite floors), paint and cigarette smoke used in residential decoration can cause indoor formaldehyde pollution in air. The active aldehyde group can directly attack the nucleophilic groups in the organism without metabolism, such as guanine, adenine, cytosine and thymine in the nucleic acid, and can covalently combine with DNA molecules to form aldehyde DNA adduct, which belong to base modified DNA damage^25^. Formaldehyde has obvious oxidative damage effect, and formaldehyde exposure will cause various oxidative damage of liver and kidney in mice, resulting in an increase of ROS and MDA content^26^. Therefore, formaldehyde was used in this study to explore the effects of oxidative damage and DNA damage by experimental mice.

Oxidative metabolism is an essential life activity of body, which inevitably produces various reactive oxygen free radicals. If the body can’t effectively remove excessive free radicals produced from the body, it will lead to oxidative damage. SOD is an active substance derived from living organisms, which can eliminate harmful substances produced from the body’s metabolism, and is the natural enemy of oxygen free radicals in the body. GR is an important cellular antioxidant, which can catalyze the reaction of oxidized glutathione (GSSG) to reduced GSH. When the body is absence of GR, it will make the cells more vulnerable to injury from oxidants. As a product of lipid peroxide, the content of MDA reflects the degree of lipid peroxidation in the body, indirectly reflects the degree of cell damage, and it has also certain cytotoxicity. The results of our study showed that the activity of SOD and GR in mice exposed to formaldehyde was low, while the content of MDA was high. With the increased dosage of pachymaran, the activity of SOD and GR increased, while the content of MDA decreased, indicating that pachymaran can enhance the activity of SOD and GR, consequently remove oxygen free radicals in the body, inhibit the oxidative damage in body, reduce the level of MDA, and shows a strong anti-oxidative damage effect. Wei et al. also found that carboxymethyl pachymaran can inhibit effectively oxidative damage induced by cyclophosphamide^27^. Some studies have found that the ability of scavenging DPPH (2,2-Diphenyl-1-Picrylhydrazyl) free radicals in pachymaran exceeds that of VitC^28^. This study further verified the anti-oxidant damage ability of pachymaran.

DNA adduct and DPCs are genetic damage markers of biomacromolecules that have attracted much attention in recent years,which represents an important genetic damage to biomacromolecules caused by environmental physical and chemical factors. The formation of DNA adduct or DPCs can affect gene expression, break the normal structure of chromosomes, and lead to the change and loss of some genetic information during DNA replication. If DNA adduct can not be repaired or repaired wrongly before cell replication, it will lead to gene mutation, cause irreversible gene damage, and then induce mutation and tumor. So it is considered that there is a certain causal relationship between the formation of DNA adduct and carcinogenesis, and DNA adduct is considered as an early detectable biomarker reflecting the process of chemical carcinogenesis. DPC was used initially to study the damage of ultraviolet and electric radiation to DNA, and then used to observe the biological effects of some anti-tumor drugs, including cisplatin. Because many environmental pollutants and chemical carcinogens, including alkylating agents, benzopyrene, arsenides, aldehydes and some heavy metals, such as chromium and nickel, can cause DPC, and the toxic effects of molecular biomarker DPC caused by exogenous chemicals have attracted much attention at present. The mechanism of DPC formation is not completely clear, which may be related to the formation of hydroxyl radicals and the reduction of intracellular sulfhydryl level. 8-OHDG is an oxidative adduct of reactive oxygen radicals attacking DNA molecules. Its detectable level can reflect the extent of oxidative damage and repair in vivo, which is of great significance for the study of degenerative diseases, aging mechanism, carcinogenesis mechanism, the relationship between environmental toxicants and oxidative stress. And, 8-OHDG can also be used to evaluate the effect of antioxidants in treating DNA oxidative damage. At present, 8-OHDG has become the most commonly applied biomarker in the study of DNA oxidative damage.

Zhang et al.found that pachymaran has the protective effect on sperm membrane and DNA in mice, and can prolong the survival time of sperm in vitro, suggesting that pachymaran may have anti-genetic damage effect^29^. In this study, our results showed that the concentration of DPC, DNA adduct and 8-OHDG was negatively correlated with the dose of pachymaran (DPC: *Spearman correlation coefficient* r_s_ = − 0.855, *P* < 0.01; DNA adduct: r_s_ = − 0.869, *P* < 0.01; r_s_ = − 0.412, *P* < 0.05). When the dosage of pachymaran is more than 2.0 g/L, pachymaran shows a good inhibitory effect on the formation of DPC and 8-OHDG, while the inhibitory effect of DNA adduct needs to reach 4.0 g/L before the effect is obvious. An obvious dose–effect relationship between pachymaran and DPC, DNA adduct and 8-OHDG has been revealed, characterized as the larger in the dose, the more obvious in the inhibition effect, indicating that pachymaran has a good anti-genetic damage effect.

## Conclusion

This study showed that pachymaran has good antioxidant and anti-genetic damage effects, and has an obvious dose–effect relationship. When the larger in the dose, the more obvious in the antioxidant and anti-genetic damage effects. As a natural immune polysaccharide, the pharmacological action of pachymaran is worthy of further development and utilization.

## Data Availability

The datasets used and/or analyzed during the current study are available from the corresponding author on reasonable request.

## Abbreviations

SOD: Super oxide dismutase
GR: Glutathione reductase
MDA: Malondialdehyde
DPC: DNA–protein crosslink
8-OHDG: 8-Hydroxydeoxyguanosine
IgG: Immunoglobulin G
IgM: Immunoglobulin M
CADM1: Cell adhesion molecule1
CCR2: Chemokine C-C-motif receptor 2
IGLL1: Immunoglobulin lambda like polypeptide1
LIGP1: Interferon inducible GTPase 1
FCGR3: Fc-gamma RIII
FCGR2: Fc-gamma RII
S100A8: S100 calcium binding protein A8
S100A9: S100 calcium binding protein A9
Chil3: Chitinase-like 3
MRP8: Myeloid-related protein-8
IFITM3: Interferon-induced transmembrane protein3
TLR4: Toll-like receptor 4
TRAF6: Tumor necrosis factor receptor-associated factor 6
NF-κB: Nuclear factor kappa-B
PKC: Protein kinase C
NF-κB: Nuclear factor-kappa B
CMP: Carboxymethylated pachymaran
TNF: Tumor necrosis factor
IκBα: Inhibitor of NF-κB-α
TNBS: Trinitrobenzene sulfonic acid
IL-33: Interleukin 33
ST2: Growth stimulating expression gene 2
S180: Sarcoma 180
H22: Hepatoma 22
NK: Natural killer cell
Bcl-2: B-cell lymphoma-2
Bax: Bcl2-associated X
Hsp90: Heat shock protein90
GSH: Glutathione
ANOVA: Analysis of variance
GSSG: Oxidized glutathione
DPPH: 2,2-Diphenyl-1-Picrylhydrazyl
ELISA: Enzyme linked immunosorbent assay
